# Low-Grade Acinic Cell Carcinoma of the Parotid Gland in a Young Adult: A Case Report

**DOI:** 10.7759/cureus.93855

**Published:** 2025-10-05

**Authors:** Jonathan McAdam, Andrew McAdam

**Affiliations:** 1 Department of Urology, Belfast Health and Social Care Trust, Belfast, GBR

**Keywords:** acinic cell carcinoma, case report, facial nerve preservation, parotid gland tumor, salivary gland malignancy

## Abstract

Acinic cell carcinoma (ACC) of the parotid gland is a rare salivary gland malignancy, typically presenting as a painless swelling. We report the case of a 32-year-old male patient who presented with a right neck swelling and was diagnosed with low-grade ACC of the superficial parotid gland. Surgical excision with total parotidectomy and facial nerve preservation was performed. Histopathology confirmed low-grade ACC without vascular or perineural invasion, and lymph nodes were negative. Postoperatively, the patient developed marginal mandibular branch weakness but remains free of recurrence under ongoing surveillance. Early diagnosis and complete surgical excision can lead to favorable outcomes in low-grade ACC, although long-term follow-up is necessary.

## Introduction

Acinic cell carcinoma (ACC) is an uncommon malignant epithelial neoplasm of the salivary glands, representing approximately 6%-10% of all parotid malignancies [1]. It is characterized by serous acinar cell differentiation and typically follows an indolent clinical course. Despite a generally favorable prognosis, local recurrence and late metastases can occur, warranting prolonged follow-up. We present a case of low-grade ACC in a young adult male, successfully treated with total parotidectomy and facial nerve preservation.

## Case presentation

A 32-year-old male patient self-referred to his general practitioner with a right-sided neck swelling of gradual onset. He denied pain, facial weakness, constitutional symptoms, or recent trauma. His past medical history was unremarkable.

**Figure 1:**
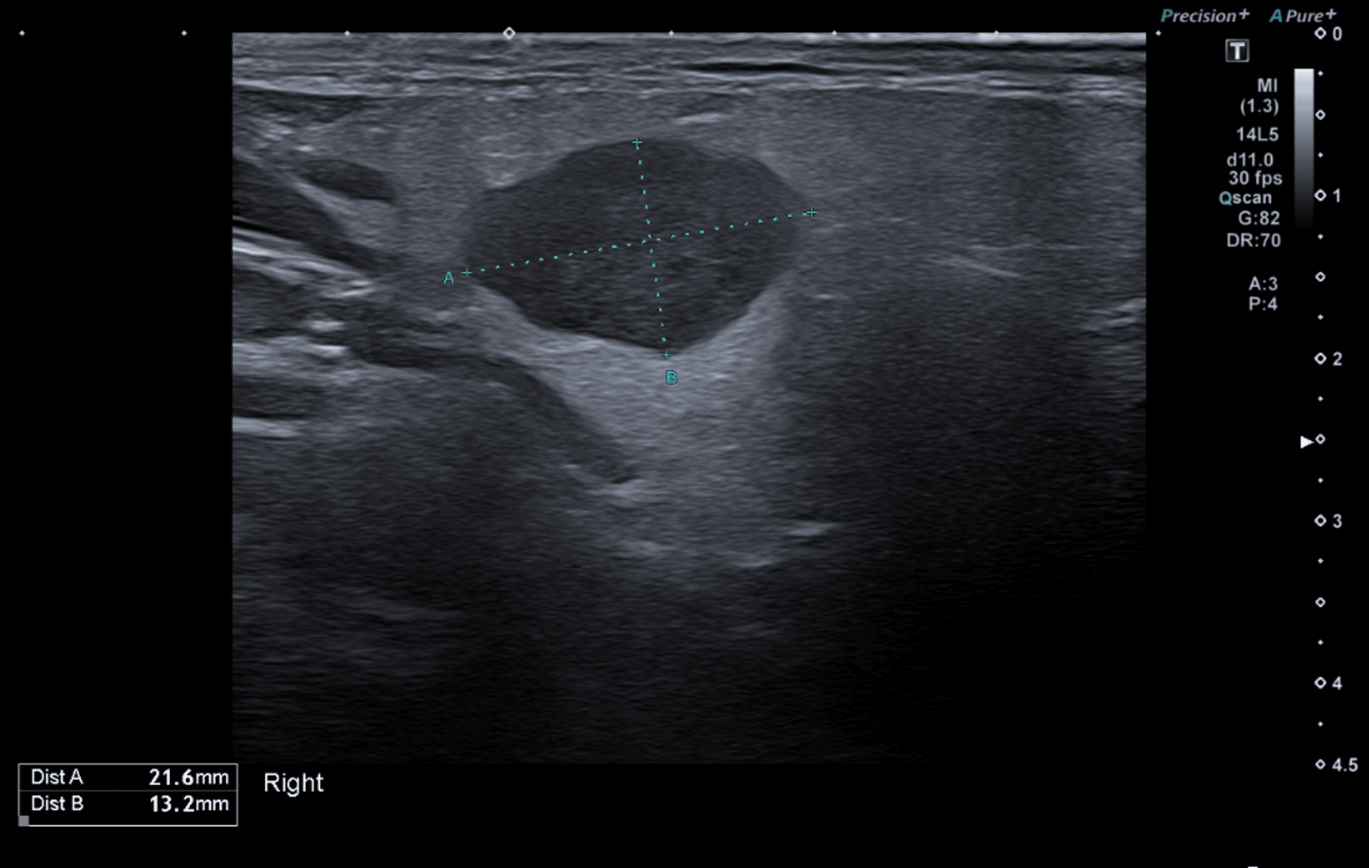
US showing parotid lesion US: ultrasound

**Figure 2:**
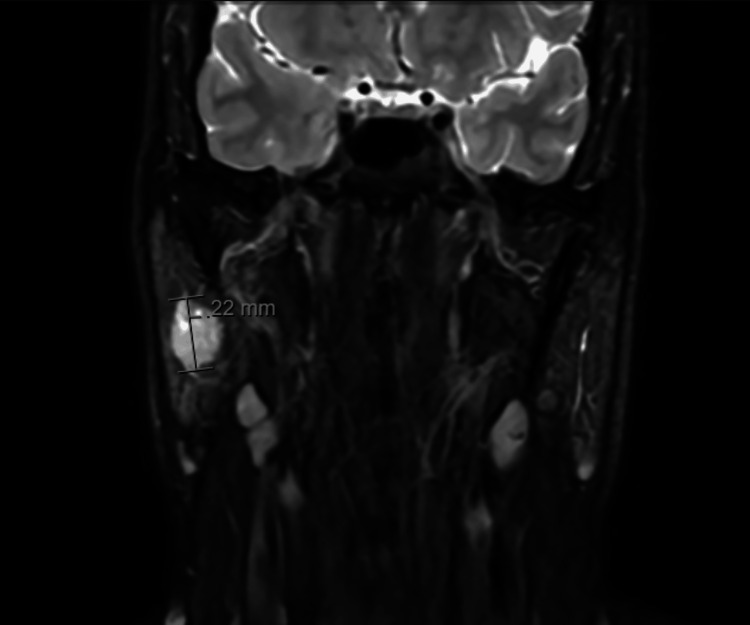
Coronal MRI neck showing parotid lesion

**Figure 3:**
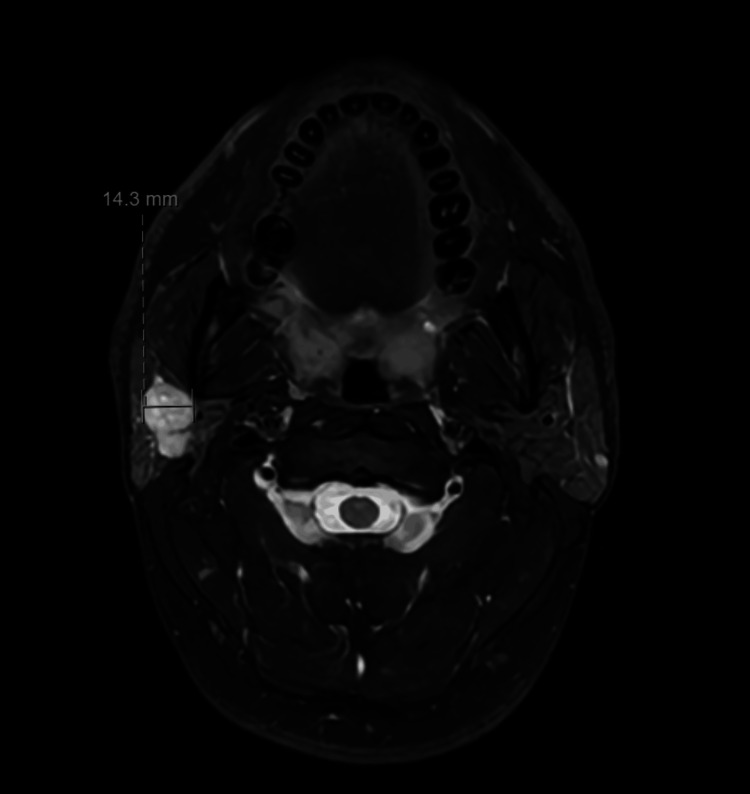
Axial MRI neck showing parotid lesion

Initial investigations included an ultrasound (US) scan, which showed a 2.6 cm lesion in the superficial lobe of the right parotid gland (Figure 1). In light of these findings, the patient progressed to have a fine needle aspiration (FNA), the cytology of which showed atypia consistent with ACC. Following these findings, the patient progressed to a staging CT neck and chest, which did not show any evidence of metastatic disease. The MRI neck (Figures 2, 3) confirmed the findings on both US and CT with a 1.9 cm lesion confined to the superficial lobe, with the facial nerve passing over the lesion and no extraparotid extension.

Initial treatment involved a total parotidectomy, with preservation of the main trunk and branches of the facial nerve. The marginal mandibular branch was noted to be stretched over the tumor but not infiltrated.

**Figure 4:**
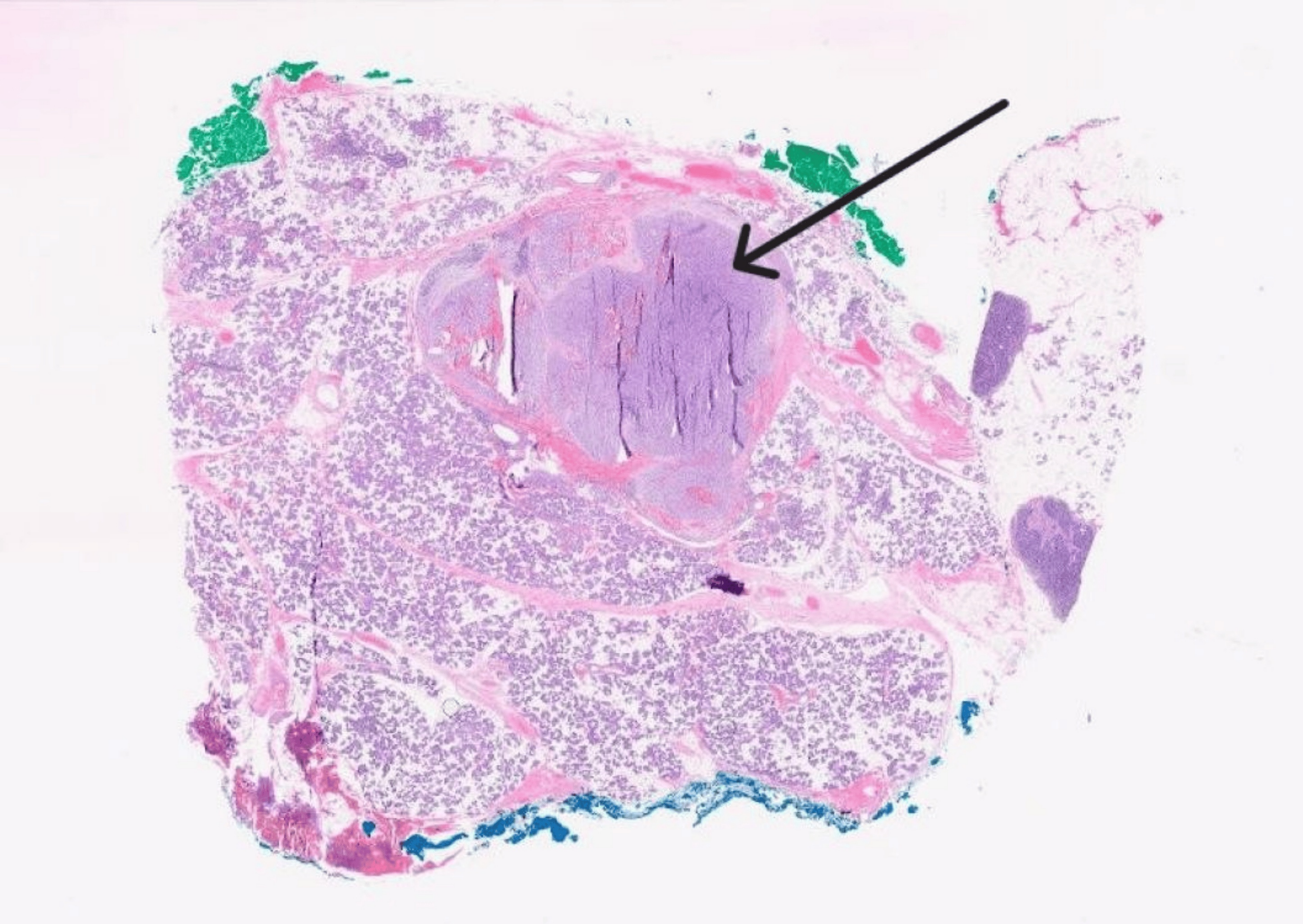
Histopathology showing encapsulated tumor (arrow)

**Figure 5:**
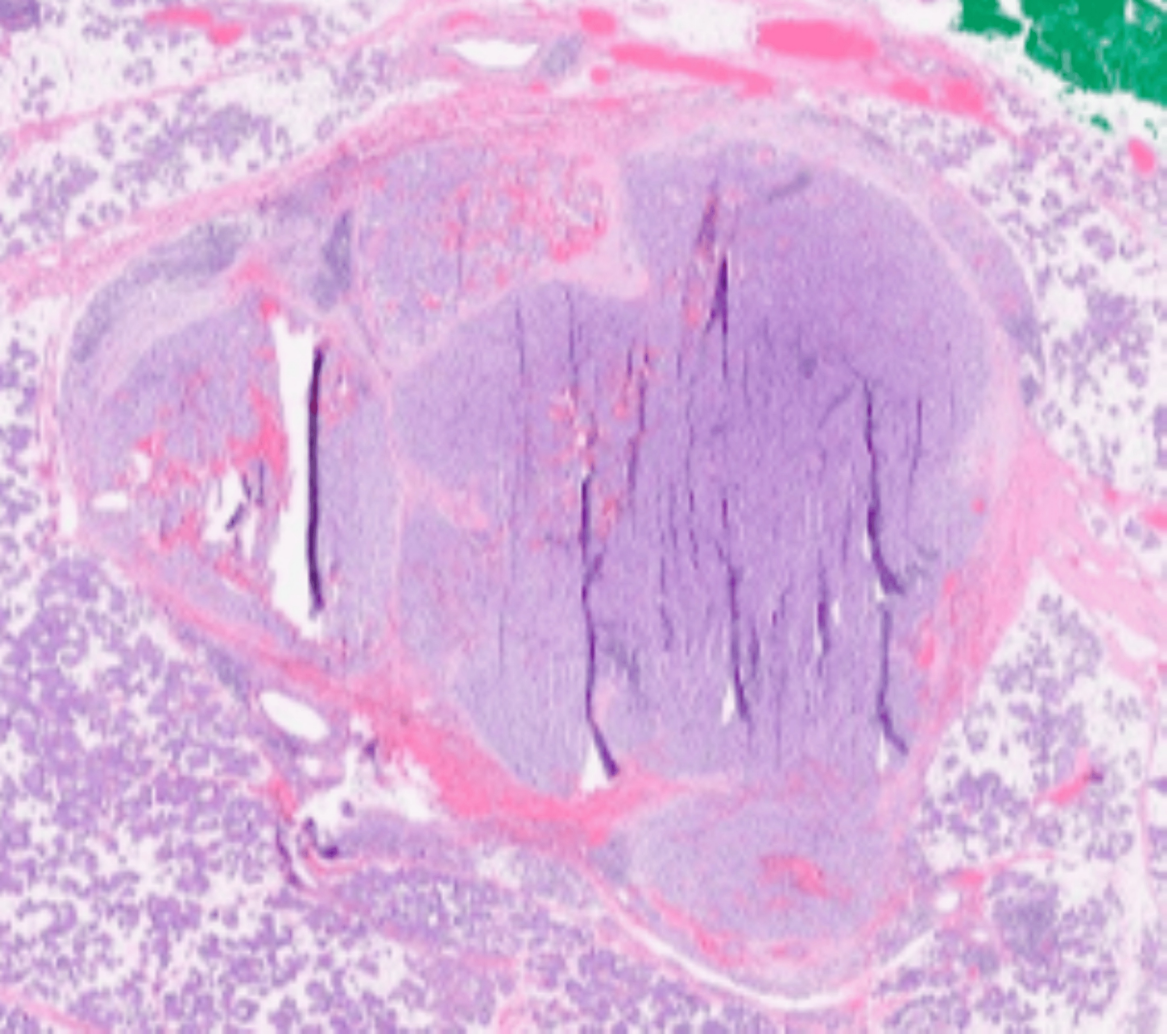
Histopathology showing tumor

Histopathology confirmed a low-grade ACC with clear margins (Figures 4, 5). The lesion was surrounded by an incomplete fibrous capsule, which had been distorted by the cautery effect. The tumor consisted of abundant serous acinar cells arranged in broad trabeculae with a focal lymphoplasmacytic infiltrate. There was no evidence of vascular or neural involvement, and the regional lymph nodes were negative for malignancy. Final staging for this case was T1N0M0.

Outcome and follow-up

The postoperative course was uncomplicated except for persistent weakness of the marginal mandibular branch, likely due to intraoperative stretching. The patient is undergoing clinical surveillance every six weeks for two years. At the most recent follow-up, there is no evidence of recurrence.

## Discussion

ACC classically presents as a slow-growing, painless parotid mass and accounts for a meaningful minority of malignant parotid tumors, with a predilection for the superficial lobe. Although it may occur across all age groups, a substantial subset of patients present in the third to fifth decades, and pediatric cases are also described, underscoring the wide age distribution. Preoperative assessment typically combines high-resolution US and cross-sectional imaging. MRI delineates tumor extent, parotid fascia integrity [1], and the relationship to the facial nerve, while CT provides complementary staging of regional and intrathoracic disease. FNA cytology is frequently informative but can be limited by sampling and overlapping cytomorphology with other salivary entities; definitive diagnosis and grading remain histopathological.

Oncologic surgery is the cornerstone of curative management. For lesions confined to the superficial lobe, either superficial parotidectomy or total conservative parotidectomy may be chosen based on tumor size, multifocality, proximity to the main trunk or branches of the facial nerve, and surgeon preference. Negative margins are a key prognostic factor [2]; positive or close margins correlate with increased risk of local recurrence. Elective neck dissection is not routinely indicated for clinically low-grade ACC because the rate of occult nodal disease is low [3]. It is considered in patients with clinically positive nodes, high-grade transformation (HGT), or advanced T category. In our case, the tumor was low-grade, located within the superficial lobe, and resected with clear margins, supporting a nerve-sparing approach [4].

The role of adjuvant radiotherapy (RT) in ACC remains nuanced [5]. Retrospective series and systematic reviews suggest that RT should be reserved for adverse features [5,6] such as T3/T4 stage disease, positive or very close margins(≤1 mm), perineural invasion, lymphovascular invasion, nodal involvement, or HGT. For early-stage, low-grade parotid ACC that is completely excised, several large series have not demonstrated a survival benefit for routine postoperative RT [5]. However, RT may reduce locoregional recurrence in selected higher risk subgroups [7]. Given the absence of adverse pathological factors in this patient, observation rather than adjuvant RT is consistent with contemporary guidance [8].

The prognosis for conventional low-grade ACC is generally favorable, with five-year overall survival commonly exceeding 90% [1] in modern cohorts. However, late events are characteristic: the median time to recurrence can approach eight years [6], and 10-year disease-free survival may fall into the 70%-80% range in historical series, highlighting the need for prolonged surveillance beyond the traditional five-year horizon. Established adverse prognosticators include positive margins [9], advanced T stage, nodal metastasis, perineural or lymphovascular invasion, and HGT. HGT, formerly termed dedifferentiation, is an uncommon but aggressive variant associated with early recurrence [10], nodal disease, and substantially worse survival; its recognition has practical implications for the extent of surgery, consideration of neck dissection, and postoperative RT [11].

The follow-up strategy should, therefore, be long-term and risk-adapted. For completely excised, low-grade, node-negative tumors without adverse features, regular clinical examination with interval imaging tailored to symptoms or equivocal findings is appropriate. In higher risk situations, including positive margins not amenable to reexcision, perineural invasion, nodal disease, or HGT, closer surveillance and a lower threshold for postoperative RT are warranted. Our patient's pathology and early postoperative course are aligned with an excellent short-term prognosis; nonetheless, we have instituted structured, prolonged surveillance to mitigate the recognized risk of late recurrence.

## Conclusions

Low-grade ACC of the parotid gland can be successfully treated with complete surgical excision and facial nerve preservation. While the histological grade and absence of adverse features indicate a favorable prognosis, vigilant surveillance is essential due to the risk of late recurrence.
